# Near-optimal experimental design for model selection in systems biology

**DOI:** 10.1093/bioinformatics/btt436

**Published:** 2013-07-29

**Authors:** Alberto Giovanni Busetto, Alain Hauser, Gabriel Krummenacher, Mikael Sunnåker, Sotiris Dimopoulos, Cheng Soon Ong, Jörg Stelling, Joachim M. Buhmann

**Affiliations:** ^1^Department of Computer Science, ETH Zurich, ^2^Competence Center for Systems Physiology and Metabolic Diseases, ^3^Department of Mathematics, ETH Zurich, ^4^Department of Biosystems Science and Engineering, ETH Zurich, ^5^Swiss Institute of Bioinformatics, Zurich, Switzerland and ^6^National ICT Australia, Melbourne, Australia

## Abstract

**Motivation:** Biological systems are understood through iterations of modeling and experimentation. Not all experiments, however, are equally valuable for predictive modeling. This study introduces an efficient method for experimental design aimed at selecting dynamical models from data. Motivated by biological applications, the method enables the design of crucial experiments: it determines a highly informative selection of measurement readouts and time points.

**Results:** We demonstrate formal guarantees of design efficiency on the basis of previous results. By reducing our task to the setting of graphical models, we prove that the method finds a near-optimal design selection with a polynomial number of evaluations. Moreover, the method exhibits the best polynomial-complexity constant approximation factor, unless P = NP. We measure the performance of the method in comparison with established alternatives, such as ensemble non-centrality, on example models of different complexity. Efficient design accelerates the loop between modeling and experimentation: it enables the inference of complex mechanisms, such as those controlling central metabolic operation.

**Availability:** Toolbox ‘NearOED’ available with source code under GPL on the Machine Learning Open Source Software Web site (mloss.org).

**Contact:** busettoa@inf.ethz.ch

**Supplementary information:** Supplementary data are available at *Bioinformatics* online.

## 1 INTRODUCTION

At the present level of development, investigations in biology require setting up complicated and expensive experiments (Kitano, 2002). Advances in measurement techniques prompted the recent growth of detailed mathematical models, which capture biological phenomena at different levels of detail. However, the employment of novel measurement techniques by itself is insufficient to achieve high predictive power. Experimental design provides the necessary guidance to determine crucial observations. Often, in fact, an important task is the selection of the most informative experiments. In systems biology, dynamical models express cause–effect relations between interacting components (Kitano, 2002). Designing optimal experiments for parameter estimation is challenging, but also well studied. At present, there already exist conclusive results and ready-to-use procedures (Bandara *et al.*, 2009; Faller *et al.*, 2003). In contrast, modern research often consists of discriminating between alternative models (Box and Hill, 1967; Kuepfer *et al.*, 2007), a task for which several questions remain open (Faller *et al.*, 2003; Kreutz and Timmer, 2009; Myung and Pitt, 2009). Design optimization for the selection of dynamic models proves especially challenging in the presence of non-linear behavior (Balsa-Canto *et al.*, 2008; Kitano, 2002). In classical statistics, ensemble non-centrality constitutes the reference technique to design experiments for model selection (Atkinson and Fedorov, 1975; Ponce De Leon and Atkinson, 1991; Skanda and Lebiedz, 2012). Recently, Bayesian techniques have been applied with success to neuroimaging and biochemical modeling (Busetto *et al.*, 2009; Daunizeau *et al.*, 2011; Kramer and Radde, 2010; Liepe *et al.*, 2013; Steinke *et al.*, 2007). Existing methods are primarily limited by computational bottlenecks, as optimization is often practically intractable.

This study introduces an efficient method to design informative experiments for selecting biological dynamical systems. Building on previous results (Krause and Guestrin, 2005), we go beyond current limitations by constructing a method that yields near-optimal combinations of time points and measurable readouts. Formal efficiency guarantees of the method are proved by reduction to a well-studied general setting (Feige, 1998; Krause and Guestrin, 2005; Nemhauser *et al.*, 1978). The method is generally applicable and has been primarily motivated by questions arising from the biological domain. We empirically evaluate the performance of the method with models of glucose tolerance and cell signaling. We apply the method to address challenging open problems of biological and medical relevance.

The manuscript is organized as follows. We start by introducing relevant facts and notions to be used in the rest of the article. Theoretical results are followed by empirical evaluation and numerical comparison with competing techniques. Finally, the method is evaluated and verified with glucose tolerance and cell signaling. Further details are presented in the Supplementary material.

## 2 BACKGROUND

We distinguish three entities: the studied system, the researcher and the measurement apparatus. The system is modeled by the researcher, who learns from the data and designs experiments by tuning the measurement apparatus. In this study, learning and reasoning follow the rules of probability theory (Baldi and Itti, 2010). Let admissible configurations of the system be called states 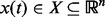. States are time-varying representations evolving over time 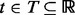. We define the ‘true model’ as 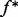, the function that governs the evolution of the system. Modeling with systems of ordinary differential equations (ODEs), we have
(1)
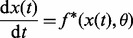

with a certain known initial condition 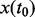. The function 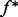 defines how infinitesimal state increments depend on current states and parameters 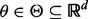 of the system. In biochemical and physiological applications, each state component quantifies molecules, concentrations or other physiological measures. In practice, parameters consist of acceptable values for reaction rates and other kinetic constants (Kitano, 2002; Zhong *et al.*, 2012). Calculating the trajectory of the system in Equation (1) from a certain starting point is an initial value problem (IVP). The ‘true model’ 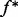 and its parameters are unknown to the researcher.

The goal of modeling is to select the most predictive model, and to estimate parameters and initial conditions. In this study, model selection is inference, that is deductive learning from data. The lack of knowledge of the researcher is not absolute. First, the researcher has access to a set of candidate models, which we call the hypothesis class 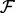. We denote a generic candidate model as 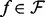. The ‘true model’ is not necessarily a candidate model available to the researcher. Let us call the scenario in which 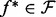 as realizable, and non-realizable otherwise. This study considers both realizable and non-realizable scenarios. Second, the researcher benefits from previous experiments, published results and domain knowledge. All these pieces of information form the *a priori* knowledge, that is the prior probability *p*( *f* ). Such probability is defined over the candidate models before observing the data.

**Fig. 1.**
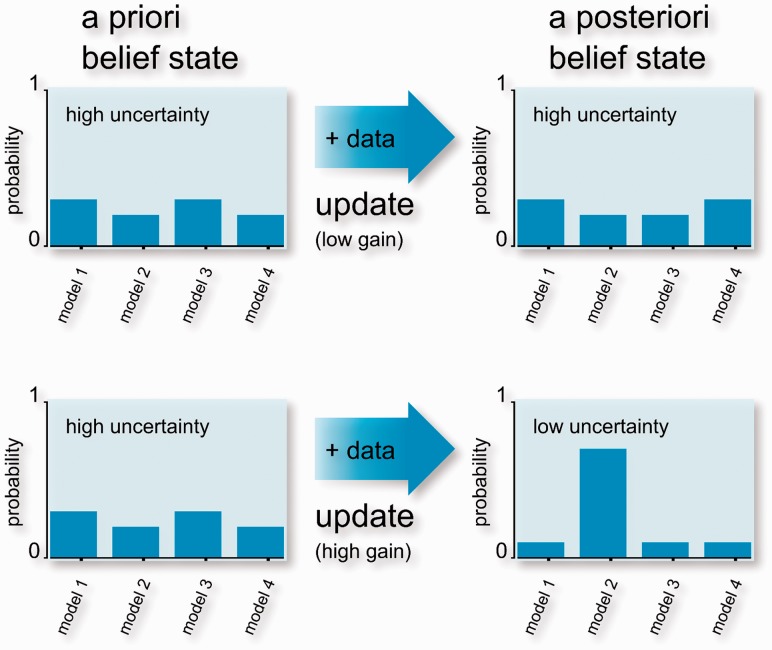
This example compares probability updates for four models. The updates are induced by two different datasets. On the top, both initial and final belief states are uninformative: the update yields low information gain. This is in contrast to the bottom plot, which shows a highly informative update: starting from an uninformative prior, the posterior concentrates the probability mass on a single model (Busetto, 2012)

Experimental measurements consist of readouts
(2)
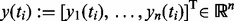

obtained through sampling. Sampling can be performed at arbitrary time points 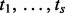. We denote the range of indexes for time points as 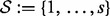 and the range for the readout variables as 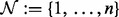, such that the index pair 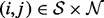 refers to the individual measurement
(3)
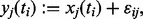

whose noise is denoted by 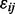. Noise terms are independent random variables sampled from known distributions *N_ij_*. Individual measurements can be grouped into datasets
(4)
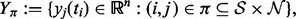

whose elements are defined by the indexes in experiment π, which is, more generally, a multiset. Adopting the Bayesian viewpoint, the researcher performs inference by calculating the probability of the models given the data, as visualized in Figure 1. The posterior probability is related to priors and likelihood through Bayes’ rule
(5)
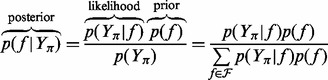

Probabilities are revised and updated for each model in 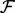 as more evidence is accumulated. The likelihood function 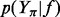 is the probability of generating a specific instance of the data with a candidate model. By construction, measurements are conditionally independent given the model, and hence the likelihood factorizes as
(6)
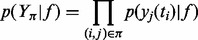

for given θ. Because of conditional independence, posteriors from previous inference are priors for subsequent experiments. This property is useful when single experiments do not yield sufficient evidence, but sequences might provide conclusive results. In practice, this advantage might prove essential to select predictive models (Xu *et al.*, 2010). Here, the primary aim is to select models, not parameters. Nonetheless, it is useful to assume a certain degree of uncertainty regarding the parameters. The model posterior is such cases obtained by marginalizing over the parameters
(7)
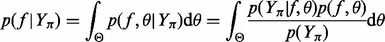

Note that models with alternative parameter values and initial conditions can be treated as alternative models. The probability of each state follows the drift equation
(8)
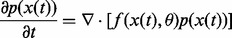

where 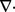 denotes the divergence operator. The equation determines the evolution over time of the state uncertainty. Conceptually, it constrains the probability of observing a certain state in the future on the basis of the dynamical properties of the system. Equation (8) can be extended to the parameter space to perform inference (Busetto and Buhmann, 2009a; Busetto *et al.*, 2009). Figure 1 schematically illustrates Bayesian inference with two updates from prior to posterior probabilities. In the example, the hypothesis class consists of 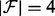 models. Informative probability distributions exhibit ‘narrow’ peaks, as they concentrate substantial mass on few models. The smaller the subset of models, the higher is the informativeness, as the data discard all other candidates. In contrast, ‘flat’ distributions indicate high uncertainty and no preference for a specific selection of models. This intuition is formalized by information theory, which offers Shannon entropy as a fundamental measure of uncertainty (Cover and Thomas, 2012).

For the purpose of learning, the researcher is not only interested in the uncertainty expressed by probabilities at a specific point in time. In contrast, the aim is to maximize the information gain, that is the additional amount of valuable information provided by new data. Figure 1 illustrates the concept with two examples. In the update on the top, the information gain is low because the posterior is almost identical to the prior. In contrast, the update on the bottom shows an informative posterior obtained from an uninformative prior. Hence, the information gain is high: the assimilated dataset yields a substantial decrease in uncertainty. At this point, the question is how to measure the gain in information. The gain yielded by a dataset is given by the relative entropy (also known as Kullback–Leibler divergence) between prior and posterior probabilities (Baldi and Itti, 2010; Liepe *et al.*, 2013)
(9)
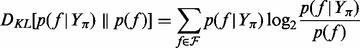



In the context of modeling, the relative entropy has a precise interpretation based on the analogy between learning and communication. The information gain corresponds to the expected number of extra bits that are lost if the dataset 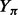 is neglected. As highlighted by the example, information gain is thus a data-dependent quantity. The example in Figure 1 shows that high gain is obtained when probabilities strongly revise the belief of the researcher, that is when extraordinary evidence is incorporated. Because it depends on the future outcome 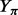 of the experiment, the gain is a quantity unknown *a priori* to the researcher. Nonetheless, prior probabilities and likelihoods are enough to predict its value in expectation. Formally, information gain can be maximized in expectation, where the expectation is taken over all possible outcomes of the experiment. To reflect the *a priori* information and the known properties of the models, information gain is weighted according to the respective measurement probabilities.

## 3 THEORETICAL RESULTS

The objective of our experimental design is to maximize the information gain in expectation, that is the mutual information
(10)
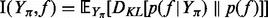

for the experiment 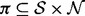. The task of optimal design is
(11)
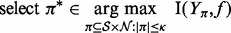

The budget 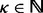 is determined by the researcher and constrains the maximum number of allowed measurements (Busetto *et al.*, 2009). In practice, the design always selects the maximum allowed number of measurements, thus justifying the choice of a limited budget. The incorporation of extra measurements, in fact, invariably adds non-negative contributions to the information obtained from the experiment. As an objective, mutual information measures the expected ability of a model to predict the data. Such an objective is not only appealing to intuition, but also theoretically justified (Cover and Thomas, 2012), and strongly supported by evidence (Baldi and Itti, 2010). The introduced method for optimal design jointly selects with π two aspects of the design: time points (when to measure) and readouts (what to measure).

The method starts by solving the IVP for each candidate model in 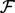. Then, it proceeds with the optimization, which consists of maximizing the objective with the maximum budget of κ measurements (Busetto, 2012). The experimental outcomes are averaged and weighted to estimate the expected information gain of the particular experiment under evaluation. For computational efficiency, optimization is performed greedily: observations are incrementally added to construct the near-optimal approximation 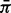 of the optimal design 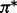. Given 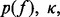 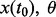, and by initializing 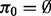, the process of optimization proceeds as follows. Iterating over *k* from 1 to κ,
(12)
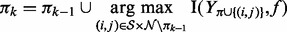

The procedure yields the final approximation 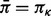 of 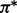. The formal worst-case performance guarantees for the method are obtained on the basis of previous results for submodular optimization in the context of active learning (Feige, 1998; Krause and Guestrin, 2005; Nemhauser *et al.*, 1978). The proof is based on a reduction to the more general setting of graphical models (Krause and Guestrin, 2005), which in turn builds on previous approximation bounds for submodular optimization (Feige, 1998; Nemhauser *et al.*, 1978).

**Theorem**

*The greedy method that selects up to κ informative readouts and time points to discriminate dynamical systems yields the near-optimal design* 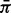 *such that*
(13)
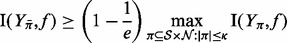

with a polynomial number of evaluations of the objective; moreover, such a constant approximation factor is the best in polynomial time, unless P = NP.

Informally, the theorem states the following: selecting the optimal experiment might be hard, and yet it is possible to easily select experiments that are provably near-optimal. It is worth noting that the yielded information is always guaranteed to be at least 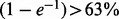 of the optimal value, that is the total experimentally achievable information. Furthermore, the empirical results introduced in the next section demonstrate that in practice, it is possible to achieve even better results in cases of concrete interest. From the computational point of view, each evaluation of the information gain requires the calculation of the posterior, which in turn requires the integral solutions of the systems of ODEs. For non-linear systems, closed-form solutions are typically unavailable (or might not even exist), thus one has to numerically approximate the solutions. Calculating the posterior is, however, as tractable as filtering for system identification. For efficiency, Sequential Monte Carlo (SMC) methods and unscented Kalman filtering may be used to perform approximate inference (Doucet and Tadić, 2003). Whereas the former technique is more general and able to deal with arbitrary multimodal distributions (Busetto and Buhmann, 2009b), the latter is particularly advantageous in the case of unimodal distributions. Approximate Bayesian computation might further extend the scope of applicability of the method (Sunnåker *et al.*, 2013b). For further details and comparison of SMC and filtering approaches, see ‘Comparison of Different Methods for Uncertainty Propagation’ in Supplementary Material.

## 4 EMPIRICAL AND APPLIED RESULTS

This section reports empirical and applied results in the domain that motivated this study: systems biology (Busetto, 2012; Hauser, 2009; Krummenacher, 2010). First, we verify the introduced method on the Bergman glucose tolerance model. We perform frequency and time point selection, showing that near-optimal solutions yield tight approximations of the global optimum (and provide similar designs, too). Second, we identify the most informative readouts to elucidate the pathway for Target-of-Rapamycin (TOR) signaling from hundreds of candidate models. Third, results are compared with other established design techniques. The results are particularly relevant to experimentalists interested in understanding metabolic control operation.

### 4.1 Dynamics of glucose tolerance

**Fig. 2.**
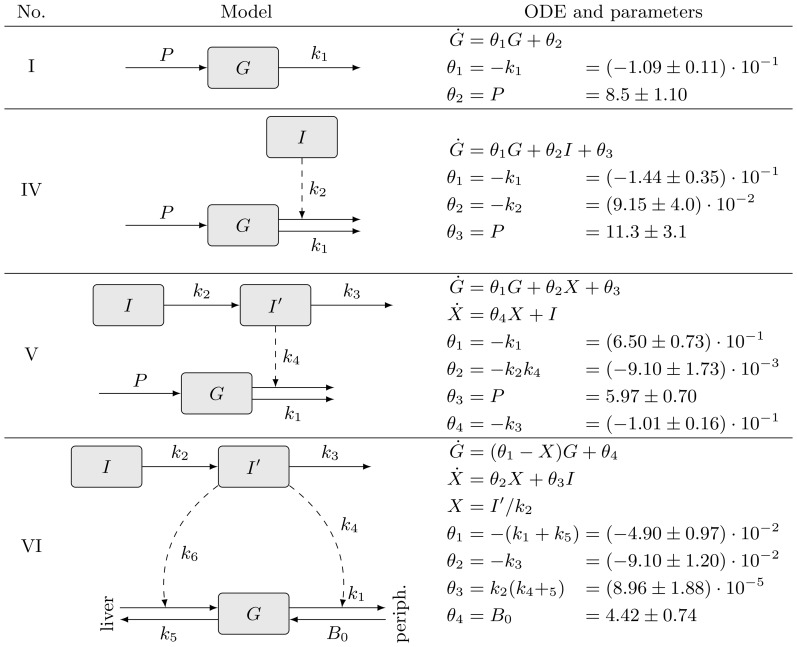
Insulin-dependent models of glucose metabolism (Bergman *et al.*, 1979). *P* is the hepatic glucose production rate. *I* is the plasma insulin concentration; its time course is not determined by the ODEs, but supplied to the models. 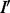 is the insulin concentration in a compartment remote from plasma. Models IV and V assume a constant production rate of glucose (*G*); in model VI, this rate is assumed to be dependent on insulin concentration. Model VI also accounts for the disappearance of glucose into peripheral tissues (‘periph.’)

**Fig. 3.**
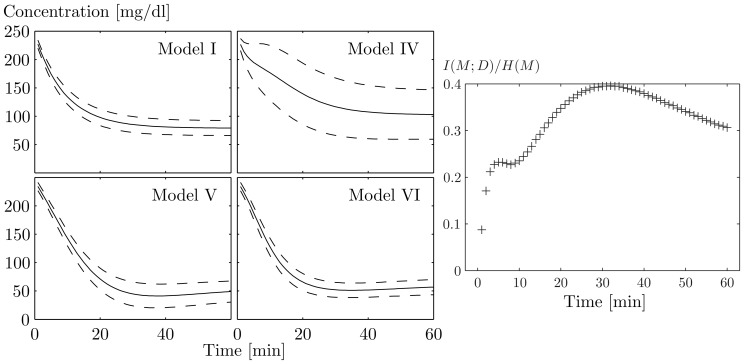
On the left, mean values (solid lines) and standard deviation of the distributions approximated by the unscented transform (dashed lines) of the glucose measurements predicted by models I, IV, V and VI. On the right, the mutual information (normalized by the entropy) for each time point

The Bergman glucose tolerance models constitute the first systematic attempt aimed at explaining the role of insulin in the degradation of blood glucose (Bergman *et al.*, 1979). This class of phenomenological models aims at identifying the mechanisms involved in reduced glucose tolerance in patients suffering from diabetes mellitus. Bergman’s models constitute a set of empirical models, regarded as the conventional reference for modeling glucose homeostasis (Kovács *et al.*, 2010). The models are highly predictive, well understood and non-linear. Figure 2 highlights the different structural properties of the models, and Figure 3 exemplifies their glucose dynamics.

**Fig. 4.**
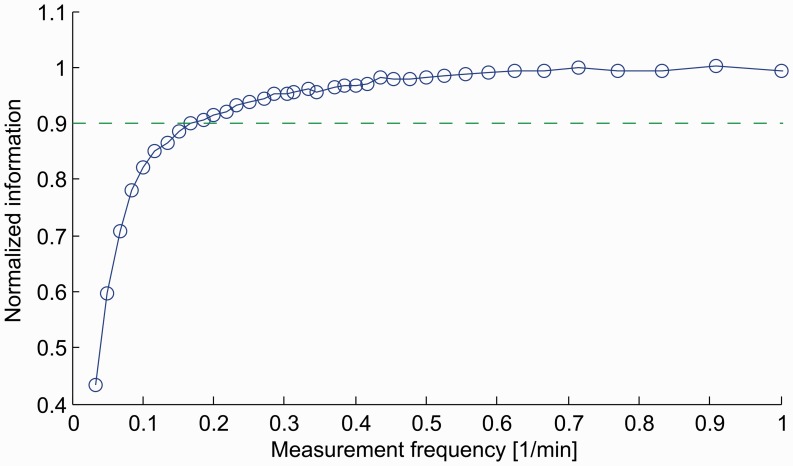
For the identification of glucose tolerance dynamics, 90% of the experimentally available information (dashed line) can be obtained with a sampling frequency of 0.2 min^−1^. Higher sampling rates yield negligible contributes to physiological modeling. Standard errors are too small to be drawn (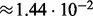 bits)

Figure 4 shows the normalized information yielded by glucose sampling frequencies in the range between 0 and 1 samples/min. More than 90% of the experimentally available information is already reachable at the uniform sampling frequency of 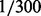 Hz (0.2 min^−1^). Also with respect to growing frequency, the mutual information follows a law of diminishing returns and, consistent with the expectation from Figure 3, it grows rapidly and then saturates. The theoretical maximum of 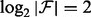 bits is rapidly approached for frequencies above 1/400 Hz (0.15 min^−1^). We also consider the case in which a glucose injection is performed as a physiological intervention. We measure the information at each individual time point to find the most informative time interval. In fact, it is possible to consider the heuristic approach of measuring with a sample frequency that is local rather than uniform and constant. The most informative region does not coincide with the beginning of the glucose degradation, but rather with the initial transition towards the steady state, as visible in Figure 3; the maximum of the information is reached at approximately 30 min from the injection. After the tipping point, the informativeness decreases while the system finally reaches the steady state. After that, residual information comes exclusively from the heterogeneous steady levels of glucose. Information is estimated with unscented propagation, which outperforms linear and SMC approximations (details in the Supplementary Material). For standard errors of 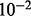 nats (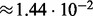 bits), the unscented approximation is between 40 and 400 times faster than that obtained with particles (which require storage and update of at least 10^4^ samples).

By selecting quintuplets from a pool of 20 time points, it is possible to estimate how close the near-optimal design is to the optimal. Optimal solutions are calculated by exhaustive search, which is extremely time-consuming, as it requires the evaluation of 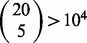 experiments. Table 1 compares optimal and near-optimal designs for 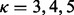. Notably, near-optimal solutions are effectively indistinguishable from the optimal ones in all cases. Not only the yielded information is practically the same (below error tolerance), but also the selections differ by a single sample over κ.

**Table 1.** Expected information gain for subsets of measurement time points of different cardinality κ for the insulin-dependent models of glucose metabolism

κ	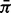 (near-optimal)	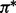 (optimal)	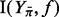	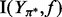
3	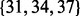	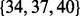	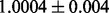	1.0009
4	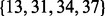	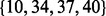	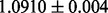	1.0940
5	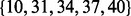	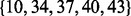	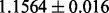	1.1585

*Note*: The measurement time points are selected from 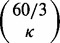 candidates from the set 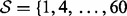}. Optimal and near-optimal solutions practically coincide.

As a consequence, optimal and near-optimal design exhibit indistinguishable probability of selecting the ‘true model’ from the data. For all practical purposes, the near-optimal selections are optimal.

### 4.2 TOR pathway

The TOR pathway is a highly conserved cell signaling structure, whose mammalian homolog is implicated in cancer, cardiovascular diseases, autoimmunity and metabolic disorders (Kuepfer *et al.*, 2007). For budding yeast, a set of 18 elementary extensions have been previously proposed in combination with a consensus core model (Kuepfer *et al.*, 2007). The elementary extensions incorporate a set of additional reactions. Combined with the core model, they represent putative mechanistic configurations of the biochemical system.

**Fig. 5.**
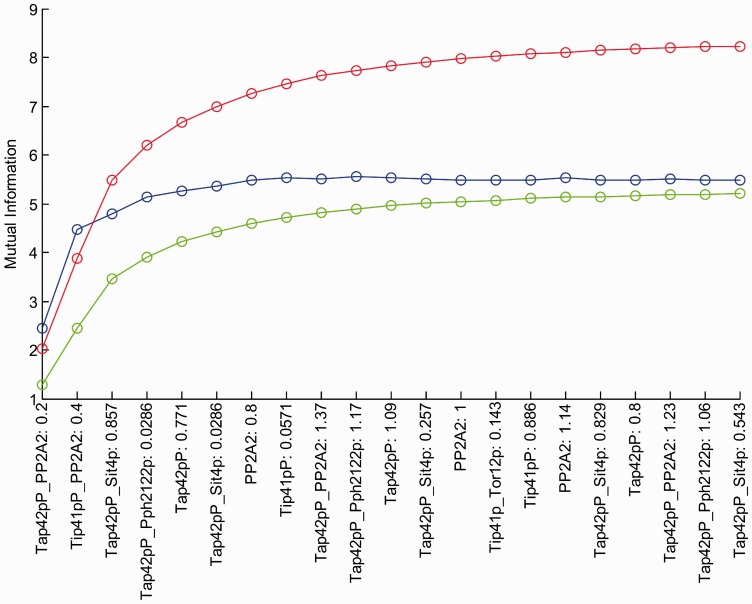
Expected information gain for increasingly large sets of selected measurements (green), each consisting of jointly selected species and time points. Online and offline bounds appear in blue and red, respectively

**Fig. 6.**
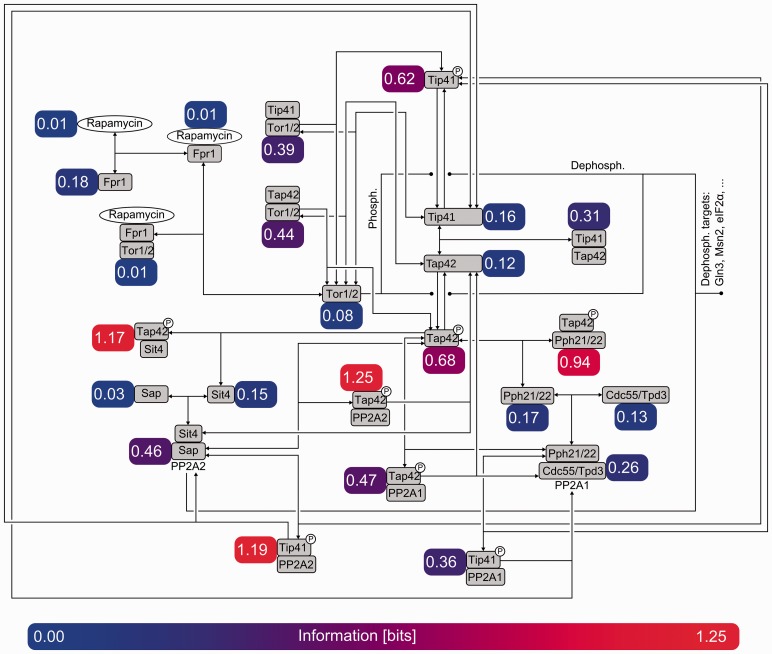
Diagram representing the individual mean mutual information over time for each chemical species in the core of the TOR pathway (Kuepfer *et al.*, 2007). Information is measured in bits and also visualized with colors ranging from blue to red

The core model consists of experimentally validated molecular interactions from inhibition of TOR kinases to the activation of protein phosphatase 2A (PP2A). In principle, the elementary extensions are not mutually exclusive (Raman and Wagner, 2011). In the evaluation, the hypothesis class 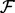 consists of 200 model prototypes. Each hypothesis corresponds to a system of ODEs with heterogeneous model complexity (from individual reactions to interlocked non-linear feedback). All 24 shared chemical species are considered measurable quantities for the experimental design. Readout selection is performed with a maximum of *s* = 50 regularly spaced time points in a relative time scale from 0 to 1.4 [time units of (Kuepfer *et al.*, 2007)]. Uniform spacing has been chosen for simplicity of description; the design method is directly applicable to any distribution of the time points. In this setting, the number of candidate experiments amounts to 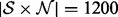. In Figure 5, the expected information gain is plotted as a function of the incremental design 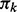 as in Equation (12), together with bounds showing tightness of approximation. The offline bound is calculated by multiplying for the approximation factor 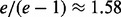 and is thus available *a priori*. The online bounds, in contrast, are iteratively calculated by using submodularity to bind the additive improvements of the objective from the current selection. The bound is
(14)
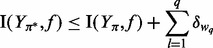

where the incremental value is 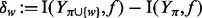 for each of the top *q* measurements *w* not considered yet (Krause and Guestrin, 1999/2007). The optimal information value is, hence, always between the achieved objective and the bound. Whereas offline bounds are trivial to compute, online bounding requires few additional calculations, but is often preferable because it yields tighter bounds. Both bounds are useful to predict quasi-plateaux of information due to saturation effects, and to evaluate the quality of the optimized (but not necessarily optimal) design (Krause and Guestrin, 1999/2007; Minoux, 1978). Tap42pP-PP2A exhibits the highest information content and is thus the best discriminative candidate. Such a species is the complex between PP2A and the phosphorylated protein Tap42p, an essential protein of the TOR signaling pathway (Düvel *et al.*, 2003). The species is known for its central role, and yet there exist substantial uncertainty regarding its precise interactions in the biochemical network (Kuepfer *et al.*, 2007). The information associated with each species is represented by Figure 6, which overlays the diagram of the core model with the mean information over time.

The theorem states that the method dominates all other efficient techniques in terms of information yield. For completeness, we also assess the performance with respect to the empirical success rate, an external score. This measure is consistent with the research goal of finding the best model and allows the comparison of the greedy approach with the available non-Bayesian alternative, that is ensemble non-centrality (Atkinson *et al.*, 2007).

**Fig. 7.**
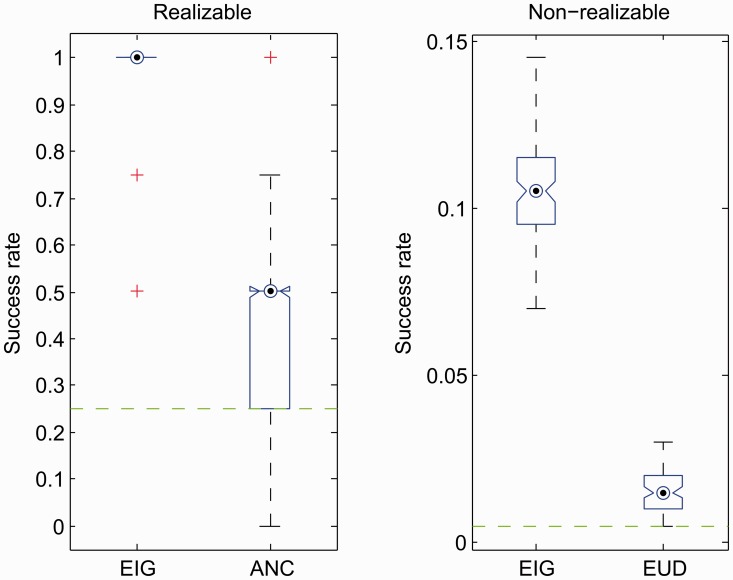
Comparison of success rates for the identification of the TOR pathway. Rates range from 0 (complete lack of success) to 1 (complete success). Realizable (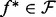) and non-realizable (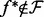) scenarios appear on left and right plots, respectively. Expected information gain, ensemble non-centrality and sum of Euclidean distances are, respectively, abbreviated as EIG, ANC and EUD. The plot on the right offers the interpretation of relative success with respect to chance (dashed horizontal line), as the maximal rate achievable for a given sample size is unknown

We evaluate the method in two benchmark scenarios: realizable and non-realizable. The success rate is the ratio of successful selections over 10^3^ runs. Model selection is considered successful when the best model is selected *a posteriori* from the data through the designed experiment. In the realizable scenario, the best model is the true model 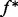, because this model is available as a candidate. In the non-realizable scenario, however, the ‘true model’ is not a candidate because 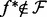. The best model then is the closest one to the ‘true model’ in terms of predictive power measured as relative entropy. In each test run, the method selects noisy readouts from the TOR models. In turn, each candidate model is assumed to generate data with additive independent normal noise (standard deviation corresponding to half of the concentration). On the left of Figure 7, near-optimal design achieves a substantially higher success rate compared with ensemble non-centrality. The evaluation highlights one of the main practical disadvantages of ensemble methods: the huge computational demands. Precisely, parameter fitting is the computational bottleneck: the step is repeated for all tested parameter configurations against what is assumed to be the correct model. Each iteration of cost minimization requires numerical solutions of non-linear ODEs, testing every model combination. This procedure is so resource-intensive that the hypothesis class has to be limited to only four models with two unknown parameters and two unknown initial conditions. The exact computational complexity of the ensemble non-centrality is unknown. However, it heavily relies on non-linear optimization, which is generally considered hard or even intractable (Nelles, 2001). It is possible, nonetheless, to calculate the number of non-linear optimization tasks involved, which follows 
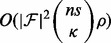
, where ρ is the number of samples employed for the approximation of the integral solution. In contrast, the greedy approach is bounded by 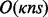 evaluations for the objective, which in turn relies on the solution of 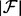 uncertainty propagation equations such as Equation (8). Combining flow propagation and Bayesian learning can be performed with the unscented Kalman filtering, which requires the solution of 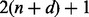 individual IVPs, where *d* is the number of free parameters in θ. This number is approximately proportional to the expected degree of the network, which follows a Zipf distribution, making it independent of network size (Szállási *et al.*, 2006). Detailed analysis and comparison with other filtering approaches is reported in Supplementary Material.

The analysis proceeds with the non-realizable scenario, which captures the fact that hypothesis classes are mere approximations of reality. Ensemble non-centrality is not directly applicable in this case because it assumes that the true model is among the candidates (and performs selections with respect to it). Taking the best approximation as the correct model, one maintains the same objective function based on the average residual sum of squares. Results are reported on the right of Figure 7 for all 200 models and 50 time points. Calculations have been performed with the submodular optimization toolbox for Matlab (Krause, 2010). As in the realizable scenario, the introduced approach yields significantly higher success rates. In contrast to the realizable case, success should be seen as a relative quantity, as the finite sample size induces an unknown scaling for the maximal rate of practical success. The results also highlight that multiple models achieve comparable predictive power and are, thus, difficult to exactly discriminate from the data.

## 5 CONCLUSION

In a complex field in which noisy data and expensive experiments constitute the norm, it is crucial to guide experimentation through rational design. Here, our main contribution is the introduction of a method that guarantees high informativeness with a polynomial number of evaluations of the information objective. The main motivation of this study is biological, but it is worth noting that the presented results for readout and time point selection are applicable to general dynamical systems. As a consequence of previous results from submodular optimization (Feige, 1998; Krause and Guestrin, 2005; Nemhauser *et al.*, 1978), we could prove that the greedy method exhibits the best constant approximation factor (unless P = NP) to design experiments for the selection among alternative dynamical systems.

This study proves that entirely rational selections can be made *a priori* with efficiency and solely on the basis of the accumulated domain knowledge. Reported results show that near-optimal experiments are effectively optimal in the application to glucose tolerance. The method outperforms the available alternatives in terms of empirical success rate, as shown for TOR modeling.

In a practical application, we used the method presented here in a study revealing nuclear phosphorylation as the key control mechanism for the transcription factor Msn2 on stress release in *Saccharomyces cerevisiae* (Sunnåker *et al.*, 2013a). By optimization of Equation (10), the experimental design was targeted to enable informative selection among 12 models representing various hypothetical mechanisms for the short-term Msn2 dynamics. In this application, the combination of experimental design and model selection led to identification, and prediction, of previously unknown and potentially generic principles for transcription factor dynamics (Sunnåker *et al.*, 2013a).

A distinct but relevant question remains open: how to reliably identify the parameters of the candidate models? This issue goes beyond the scope of this study, as it strictly belongs to the domain of system identification (Busetto and Buhmann, 2009a). At the same time, it is an aspect that deserves special attention, as design and modeling are part of the same hypothetico-deductive process. We conclude that the introduced method may be useful to guide intuition through quantitative indicators and thus accelerate scientific discovery.

## Supplementary Material

Supplementary Data
