# Supplementary material for: Near-optimal experimental design for model selection in systems biology
Source: Bioinformatics. 2013 Jul 29;29(20):2625–32. doi: 10.1093/bioinformatics/btt436 (PMC3789540; doi:10.1093/bioinformatics/btt436)
Supplement: Supplementary Data [file supp_29_20_2625__index.html]

Near-optimal experimental design for model selection in systems biology — Near-optimal experimental design for model selection in systems biology — Supplementary Data 

# Near-optimal experimental design for model selection in systems biology

## Supplementary Data

files

**Files in this Data Supplement:**

- Supplementary Data - pdf file
- Supplementary Data - png file
