# Supplementary material for: Near-optimal experimental design for model selection in systems biology
Source: Bioinformatics. 2013 Jul 29;29(20):2625–32. doi: 10.1093/bioinformatics/btt436 (PMC3789540; doi:10.1093/bioinformatics/btt436)
Supplement: Supplementary Data [file supp_btt436_SI.pdf]

Supplementary Information of

"Near-optimal Experimental Design for Model Selection in Systems Biology"

Alberto Giovanni Busetto [1,2,*], Alain Hauser [3], Gabriel Krummenacher [1], Mikael Sunnåker [2,4,5], Sotiris Dimopoulos [2,4,5], Cheng Soon Ong [6], Jörg Stelling [4,5], Joachim M. Buhmann [1,2]

[1]Department of Computer Science, ETH Zurich

[2]Competence Center for Systems Physiology and Metabolic Diseases, Zurich

[3]Department of Mathematics, ETH Zurich

[4]Department of Biosystems Science and Engineering, ETH Zurich

[5]Swiss Institute of Bioinformatics, Zurich

[6]National ICT Australia, Melbourne

1 Application to Biochemical Reaction Networks

The aim of the introduced method for experimental design is that of model discrimination. In its application to biochemical reaction network modeling, each element of the hypothesis class $\mathcal{F}$ consists of an alternative reaction network. Such hypotheses offer hypothetical explanations for the studied biochemical process. Models are identified with functions $f \in \mathcal{F}$, which define vector fields for a set of biochemical reactions. The kinetics of the system is often described in terms of the ODE system

$$\frac{\mathrm{d}x(t)}{\mathrm{d}t} = Nr(x(t), \theta) \equiv f(x(t), \theta), \tag{1}$$

where $x(t) \in \mathbb{R}^n$ is the vector of concentrations of the chemical species involved in the model, $r(x(t), \theta)$ is the vector of reaction rates for species concentrations $x(t)$ and kinetic parameters $\theta \in \mathbb{R}^d$, and N is the stoichiometric matrix mapping the q reactions to the n state component [1]. Since the integral solution of the system of ODEs is not only parameterized by the kinetic parameters, but also by initial conditions, the total parameter of the model is defined as the compound vector

$$\xi = \begin{bmatrix} x(t_0) \\ \theta \end{bmatrix}. \tag{2}$$

Furthermore, f is assumed to be differentiable with respect to $x(t)$ and θ, such that the preconditions for the Picard-Lindelöf theorem are fulfilled and such that Eq. (1) has a unique solution. The IVP is assumed to be well posed in the sense of Hadamard.

2 Uncertainty Propagation without Perturbations

This section presents different methods for the numerical calculation and the approximation of the factorial likelihoods $p(f|Y_\pi)$. The process is often referred to as uncertainty propagation since the calculations consist of propagating the probability distribution reflecting uncertainty regarding the values of the total parameter ξ.

Forward equations. Let $p(t; x, \theta)$ be the probability density of a system described by the system of ODE at time t for concentration x and kinetic parameters $\theta \in \mathbb{R}^d$. In particular, $p(t_0; x, \theta) = \mathcal{N}(\xi; \mu_\xi, \Sigma_\xi)$ because of the assumption in the distribution of the parameters. By solving the partial differential equation (PDE) given by the continuity equation for $p(t; x, \theta)$,

$$\frac{\partial}{\partial t} p(t; x, \theta) = -\operatorname{div}_x[p(t; x, \theta) f(x, \theta)]$$

and integrating out the resulting probability density over θ, one gets the probability density $\int p(x, \theta) \mathrm{b}\theta$ of the state of the system at any time point and could calculate the probability density of measurement data out of that. The numerical solution of PDEs becomes, however, harder with increasing dimension of the surrounding space. The straightforward application of this approach does not seem to be suited for the solution of typical problems from systems biology with, for instance, $\gtrsim 50$ species and comparable numbers of kinetic parameters. Feasible alternatives which overcome the mentioned limitation are described below.

Sequential Monte Carlo methods aim at approximating a probability density through a discrete probability measure defined on a set of random samples or representatives of the original measure together with associated weights [2]. In the considered cases, samples of the total parameter ξ are drawn in order to approximate the integral form of the likelihood function by a sum over parameter samples.

The approximation resulting from the linearization of the flow with respect to the total parameter ξ suggests that the mean of the concentration distributions at different times is propagated by the flow ϕ. For the nonlinear system of ODEs f, this is in general not true. A better approach for uncertainty propagation is given by the so-called unscented transform [3]. Linearization techniques for uncertainty propagation have a long tradition in filtering techniques and are part of the Extended Kalman Filter (EKF); the unscented transform has been

developed to overcome the known problems of the EKF when applied to strongly nonlinear systems [2, 3]. While giving better results for nonlinear systems, it still allows efficient calculation and the assumption of normal distributions – a set of properties making it a good choice for the calculations of the mutual information. The unscented transform is only suited to approximate transformations between two probability spaces of the same dimension. The linearization of the flow and the unscented transform both approximate the probability distributions by normal or Gaussian ones, while the particle method does not. Therefore we will denote the former ones as Gaussian methods to distinguish them from the (more general) particle method.

3 Comparison of Uncertainty Propagation Methods

First, we demonstrate the different methods for uncertainty propagation presented in Section 2: particle method, linearization of the flow and unscented transform. We are only interested in probability distributions of measurements at single time points, neglecting correlation effects. Our main aim is to compare quality and computation time of the different propagation methods:

- How fast does the density approximated by the particle method converge to the true distribution?
- How well is the true distribution of measurements approximated by the Gaussian methods, the linearization of the flow and the unscented transform? Is it safe to use a Gaussian method instead of the particle method able to approximate *any* probability distribution to a given accuracy?
- What is the memory and time consumption of the different propagation methods? Is it worth to use Gaussian methods instead of the particle method?

In contrast to Gaussian methods, the particle method is in principle able to handle any distribution to an arbitrary precision by calculating with a large number of particles. Therefore its results can be used as a reference for comparative evaluations. For this aim, the particle method is executed several times by gradually increasing the particle number z until convergence is observed. The distribution gained that way is compared to the normal distributions calculated by the Gaussian methods. Normal distributions are completely determined by their mean and covariance (or variance, resp., in the one-dimensional case). The comparison of mean and variance of the distributions calculated by the Gaussian methods to those of the reference distribution can therefore be used to measure the quality of the Gaussian approximations. For the comparison of the means, we introduce their **relative errors**:

$$e := \frac{|\mu_{Gaussian} - \mu_{PI}|}{\sigma_{PI}},$$

where $\mu_{Gaussian}$ is the mean calculated with one of the Gaussian approximations, μ_{PI} and σ_{PI} are mean and standard deviation calculated by the particle method.

We here bound the computational and storage complexity of alternative filtering approaches. In the table, m denotes the number of parameters, k the number of particles. PC denotes the particle method with likelihoods approximated by a Gaussian mixture model, LC is the local linearization of the flow, LI the linearization with independent measurements, and UI Unscented Kalman filtering.

Propagation method	Number of ODEs	Memory usage
PC	kn	kns
LC	$n(n+m)$	$ns+(ns)^2$
LI	$n(1+n+m)$	$ns+n^2s$
UI	$2(n+m)+1$	$ns+n^2s$

All experiments are performed with Matlab 7.8 under Linux on an Intel Core Duo machine with a clock frequency of 3.16 GHz and 2 GB of RAM and Brutus.

3.1 Theorem and Proof

Theorem. *The greedy method which selects up to κ informative readouts and time points to discriminate dynamical systems yields the near-optimal design $\bar{\pi}$ such that*

$$\mathrm{I}(Y_{\bar{\pi}}, f) \geq \left(1 - \frac{1}{e}\right) \max_{\pi \subseteq \mathcal{S} \times \mathcal{N} \colon |\pi| \leq \kappa} \mathrm{I}(Y_\pi, f), \quad (3)$$

with a polynomial number of evaluations of the objective; moreover, such constant approximation factor is the best in polynomial time, unless P=NP.

Proof. For each $f \in \mathcal{F}$, the trajectory in the state space is determined by the integral solution of the IVP for the system of ODEs. Let each integral solution be $\phi_f(t)$, such that $\phi_f(t) = x(t)$ for all $t > t_0$, for the respective systems of ODEs in the hypothesis class $\mathcal{F}$. On the basis of the measurement model defined the main text, it is possible to construct the graphical model with the set of nodes $\mathcal{V} = \{f\} \cup \{y_j(t_i)\}_{(i,j)\in\pi}$ and the set of directed edges $\mathcal{E} = \{(y_j(t_i)|f) \colon (i,j) \in \pi\}$. The edges express the conditional independence of each $y_j(t_i)$ given f. The set of nodes $\mathcal{V} = \{f\} \cup \{y_j(t_i)\}_{(i,j)\in\pi}$ can then be partitioned into two disjoint subsets $\{f\}$ and $\{y_j(t_i)\}_{(i,j)\in\pi}$. In these partitions, the random variables in $\{y_j(t_i)\}_{(i,j)\in\pi}$ are conditionally independent given the integral solution $\phi_f(t)$, which in turn depends on f. Such configuration is guaranteed to yield a mutual information as a function of the experiment π which is submodular, non-decreasing and for which $\mathrm{I}(Y_\emptyset, f) = 0$ [7]. At this point, our

Propagation method	Model	$t = 10$ min		$t = 20$ min		$t = 50$ min	
		mean	SD	mean	SD	mean	SD
Particles	I	134.4	13.2	97.7	14.3	79.6	13.3
	IV	176.2	47.3	138.9	51.3	103.1	43.4
	V	143.2	13.3	71.2	19.0	45.1	19.1
	VI	127.7	15.0	65.9	14.8	54.3	13.0
Linearization	I	133.8	13.2	96.8	14.1	78.7	13.0
	IV	171.3	45.2	130.5	44.6	95.4	32.9
	V	142.8	13.3	70.7	18.8	45.7	18.1
	VI	127.0	18.7	64.3	21.1	53.2	24.8
Unscented transform	I	134.3	13.3	97.8	14.4	79.5	13.3
	IV	176.5	48.3	139.5	54.1	104.1	44.8
	V	143.1	13.4	71.1	19.1	45.0	19.7
	VI	127.7	15.1	65.8	14.9	54.3	13.0

setting inherits the $(1 - 1/e)$ factor in the approximation bound with a polynomial number of evaluations [7, 9], which is the best factor unless P=NP [7, 10] □.

3.2 Results

The four different Bergman models (I, IV, V and VI) predict a similar glucose time course; model IV has a notably larger uncertainty than the other models. All models show a rapid glucose degradation in the first 20 minutes and then reach a steady state. The mean blood glucose concentration in this steady state lies between 50 mg/dl (models V and VI) and 100 mg/dl (model IV). The uncertainty generally increases during the degradation phase, has a maximum around the point where the systems enter steady state, and then slightly decreases again for most of the models. This behavior is pronounced in model IV.

The distributions calculated by the particle method converge to the true ones when using more and more particles. When using 10^2 particles, even narrow distributions are not represented well; with 10^3 particles, narrow distributions are well represented, whereas broad ones (e.g. model IV) are still quite fuzzy, even around their center; with 10^4 particles, the distributions are already quite precise. In the following, we will use the densities calculated by the particle method with $z = 10^5$ particles as a reference to evaluate the normal approximations calculated by the Gaussian methods; this distribution gives a good approximation to the true ones.

The measurement distributions calculated by the Gaussian methods are similar to the reference distribution calculated by the particle method (table below and Fig. 3). Mean and standard deviation of the concentration distributions calculated by different methods are reported in the table below.
The relative errors e of the means calculated by the linearization of the flow are mostly below 11%; only in two cases, both for model IV, the one having the

highest uncertainty (Fig. 1), the mean deviates from the reference distribution by around 0.17 standard deviations. The unscented transform performs significantly better; even for model IV, the relative error is below 2.5%, and in all other cases even below 1% (see table). The larger error for model IV is probably due to the skewness of the true distribution (Fig. 3) that that gets lost when approximating with a (symmetric) normal distribution.

The standard deviations calculated with the linearization of the flow match the reference deviations calculated using the particle method with a difference of less than 5% in models I and V. The standard deviations of models IV and VI differ much more from the reference, namely between 5% and 92%. As well as for the means, the unscented transform performs better in approximating the standard deviations. With only one exception (model IV at time $t = 20$ min), all standard deviations from the unscented transform do not differ by more than 5% from the reference values.

On our test machine, the calculation of the approximation using the particle method took 0.54 s per 1000 particles using the fast ODE solver `CVODE` [4] linked with the Systems Biology Toolbox 2 [5]. The calculation of the densities with the linearization of the flow took 0.17 s using Matlab's ODE solver `ode15s`, with the unscented transform 0.48 s using `ode15s` or 0.013 s using `CVODE`.

For the Bergman minimal models, and under the assumption of independent measurement values, the method of choice for uncertainty propagation is clearly the unscented transform.

Although the linearization of the flow gives results of inferior quality compared to those of the unscented transform, the overall judgment for the unscented transform given above is also true for this method. Nevertheless, the unscented transform is clearly preferable when the assumption of independent measurement values is made; without that assumption, linearization of the flow is still a valuable alternative to the particle method. More caution has to be taken in the case of strong nonlinearity of large uncertainty; since the linearization of the flow operates strictly *locally*, calculating the flow and its derivatives solely for the mean total parameter μ_ξ, it cannot average out local nonlinearity as the unscented transform does.

Despite the good performance of the Gaussian methods, we want to emphasize that the results gained here for the Bergman models cannot be transferred to any other set of systems biological models. Gaussian methods can e.g. not at all handle systems that exhibit bifurcations where a unimodal distribution can be propagated into a bimodal one [1]. We therefore propose the following proceeding when faced with an experimental design problem for a new set of systems: first, propagate the uncertainty with a low number of particles. By a "low number", we mean a number that does not slow down calculation too much, but nevertheless is sufficient to recognize important properties of the measurement distributions. If they are seen to be unimodal and not too skew, a Gaussian method is appropriate for further investigations; if the assumption of independent measurement values are made, the unscented transform is to be preferred, otherwise the linearization of the flow.

The comparison of the different computation times shows that the number

of ODEs that have to be solved for the different models does not determine the computation time alone. For both Gaussian methods, the number of ODEs to be solved for the Bergman models is approximately the same, 37 for the linearization of the flow and 42 for the unscented transform. Nevertheless, the computation time for the unscented case is approximately three times higher than for linearization of the flow using the same ODE solver `ode15s`. A possible explanation for this is that the ODEs of the derivatives of the flow are easier to solve than those of the flow itself, what is plausible at least for mass action kinetics, or that the calculation of the sample points for the unscented transform (Section 2) involving a Cholesky decomposition also takes a considerable amount of time. The latter guess is substantiated by the comparison of the computation times of particle method and unscented transform. For $z = 10^3$ particles, a total of $6 \cdot 10^3$ ODEs have to be solved with the particle method for all four models, about 140 times more than for the unscented transform. Nevertheless, the computation times only differ by a factor of approximately 40.

4 Bergman Minimal Models

The Bergman Minimal Models are a small set of of simple models of glucose degradation, to estimate insulin sensitivity. They regard the time course of insulin concentration as input and the glucose concentration as output of the system. In addition to those two species, they include no more than five parameters. Because of this, calculation with the models is quick. For three of the seven original models [12], not all of the parameters were identifiable, the four remaining ones are used for testing.

The four models used for the evaluations are shown in Fig. 4.

For the simulations, all the models are assigned a prior probability of $p(f) = \frac{1}{4}$ and measurement uncertainty of $\Sigma_v = 5(mg/dl)^2$ is used. This is 2% of of the glucose concentration in the steady state.

4.1 Experiments

When we let the algorithm select a set of two measurement time points, the mutual information between data and models is naturally higher. Having more measurements will increase (or at least not decrease) our information about the models. Figure 5 shows the expected information gain for measuring the glucose concentrations at two time points. The maximum information can be gained when measuring at 32 minutes and at 33 minutes, marked by the blue cross in the plot.

It might seem surprising that two measurements so close to each other lead to a higher mutual information than for example measuring at the beginning and at the end of the time course. But by having two adjacent measurement values, information about the first derivative of the curve can be obtained.

The greedy optimization algorithm, only approximately finds an optimal set of measurements. Because of submodularity of expected information gain

we know, that we are at least 63% close to the optimum, but this is a very loose bound. Therefore it is interesting to see how close we actually are to the optimal solution. Since the Bergman minimal models are quick to calculate and if we only consider a set of 20 time points to select from it was possible to compute the expected information gain for all $\binom{20}{5} = 15504$ combinations of five measurements.

The expected information gain is shown in Tab. 1 in the main text for all possible subsets of cardinality $\kappa = 3, 4, 5$. The expected information gain for the optimal and near-optimal subsets is very close and within expected error. The subsets also only differ in one measurement time point.

5 TOR Models

In the TOR pathway, type 2A phosphatases (PP2As) control various cellular functions. The phosphatases are modulated by the protein kinases Tor1p and Tor2p. Rapamycin inhibits the TOR kinases which then leads to PP2A activation. Tap42p and Tip41p regulate both the kinase and the phosphatases. The core model of TOR signal transduction summarizes all available knowledge [14]. But several aspects of the pathway are still unclear. Therefore 18 sets of reactions, that represent hypothetical biochemical features, were developed. These extensions together with the core model were then used to do model selection [14].

The set of models that we consider consists of all plausible combinations of the core model and the 18 extensions mentioned above. In principle, 2^{18} combinations are possible. After the removal of biochemically inconsistent hypotheses, there remain 69′120 candidate models.

5.1 Experiments

The models used for testing were selected like this: First we used Matlab's K-means algorithm to cluster the whole set of models into 200 clusters. Then the models closest to each of the cluster centers were chosen as representative models for that cluster.

- All of the TOR models share the first 24 chemical species, those are used as possible *measurement species*.
- After 0.7 seconds the concentrations of all the chemical species reach steady-state, to be on the save side we use 50 *time points* between 0s and 1.4s.
- To compute the integral in the Kullback-Leibler divergence, 10^4 Monte Carlo samples are used.
- Since we don't have any prior information on the probability of the models, we use a *uniform prior*.

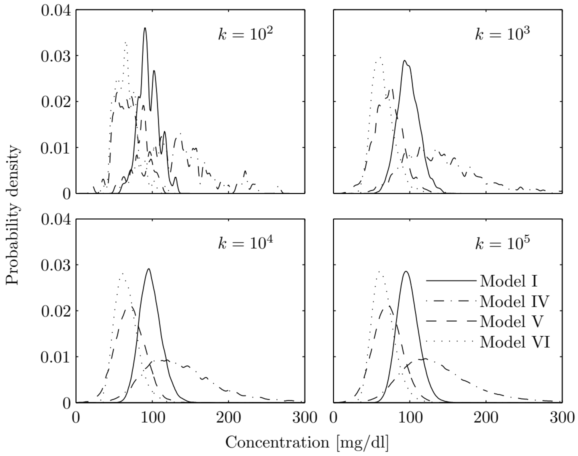

Figure 1: Probability distributions of glucose concentration measurements at $t = 20$ min predicted by the particle method, for different particle numbers k.

This leads to a set of 1200 different combinations of measurement species and measurement time points. We use a parallel implementation to compute the unscented transform and the mutual information. The lazy greedy algorithm [15] is used to do the optimization.

Figure 4 shows the first twenty one most informative measurement time points and species with the corresponding expected information gain for conducting the measurements up to that measurement. The blue line shows the online bound and the red line the offline bound for the expected information gain. Even tough the offline bound on expected information gain is quite loose, we can see that we are close to the optimum, because the expected information gain is coming close to the online bound.

References

[1] Z. Szallasi, J. Stelling, and V. Periwal, System modeling in cell biology: from concepts to nuts and bolts, *The MIT Press*, Cambridge, Massachusetts, 2006.

[2] M. S. Arulampalam, S. Maskell, N. Gordon, and T. Clapp, Tutorial on Particle Filters for Online Nonlinear/Non-Gaussian Bayesian Tracking, *IEEE Transactions on Signal Processing*, 50:174–188, 2002.

[3] S. J. Julier and J. K. Uhlmann, New extension of the Kalman filter to nonlinear systems, *Signal Processing, Sensor Fusion, and Target Recognition VI*, volume 3068, 182–193. SPIE, 1997.

[4] S. D. Cohen and A. C. Hindmarsh, CVODE, a stiff/nonstiff ODE solver in C, Computers in Physics, 10(2):138–143, 1996.

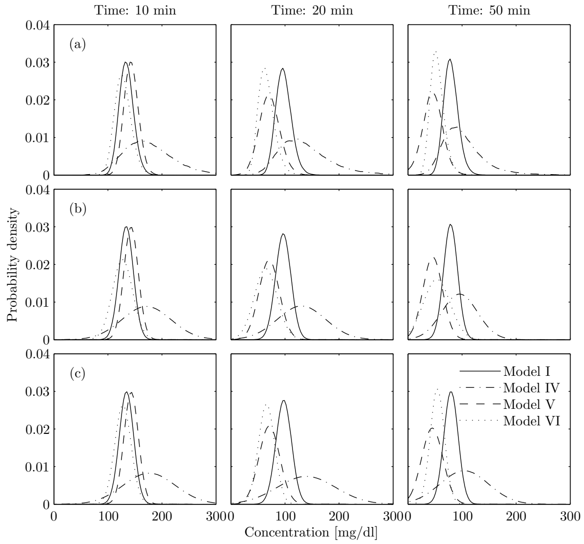

Figure 2: Probability distributions of glucose concentration measurements predicted by the Bergman models calculated with different uncertainty propagation methods: (a) Particle filter with $z = 10^5$ particles (b) Linearization of the flow (c) Unscented transform. The three time points chosen represent the different phases of the the evolution of the glucose level (Fig. 1): the first one lies in the degradation phase, the third one in the steady state phase, and the second one in the transition phase between degradation and steady state.

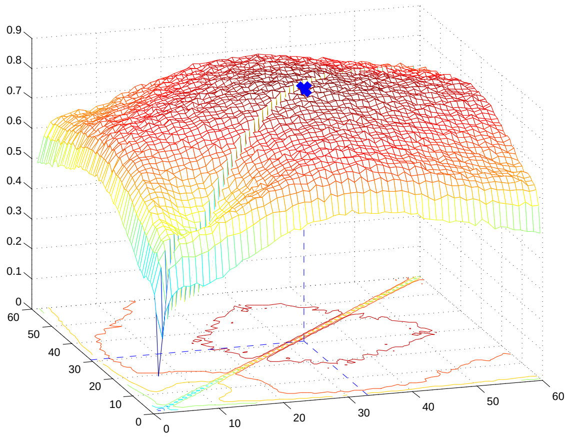

Figure 3: Mutual Information for two measurement time points of glucose in the Bergman models.

[5] H. Schmidt and M. Jirstrand, Systems Biology Toolbox for MATLAB: a computational platform for research in systems biology, Bioinformatics, 22(4):514–515, 2006.

[6] H. Jeong, B. Tombor, R. Albert, Z. N. Oltvai, and A.-L. Barabasi, The large-scale organization of metabolic networks, Nature, 6804:651–654, 2000.

[7] A. Krause and C. Guestrin, *Near-optimal Nonmyopic Value of Information in Graphical Models*, Proceedings of the 21st Conference on Uncertainty in Artificial Intelligence, 2005.

[8] A. Krause and C. Guestrin, *Near-optimal Observation Selection Using Submodular Functions*, Proceedings of the 22nd Conference on Artificial Intelligence, 1650–1654, 2007.

[9] G. L. Nemhauser, L.A. Wolsey, and M.L. Fisher, An analysis of approximations for maximizing submodular set functions - I, Mathematical Programming, 14:265–294, 1978.

[10] U. Feige, *A Threshold of Ln n for Approximating Set Cover*, Journal of the ACM 45(4), 634–652, 1998.

[11] M. Minoux, Accelerated greedy algorithms for maximizing submodular set functions, Optimization Techniques, Springer Berlin / Heidelberg, 7:234-243, 1978.

[12] R.N. Bergman, Y.Z. Ider, C.R. Browden, and C. Cobelli, Quantitative Estimation of Insulin Sensitivity, *American Journal of Physiology 236(6)*, G667–G677, 1979.

[13] H. Kitano, A. Funahashi, Y. Matsuoka, and K. Oda, Using process diagrams for the graphical representation of biological networks Nature biotechnology, Nature Publishing Group, 23:961-966, 2005.

[14] L. Kuepfer, M. Peter, U. Sauer, and J. Stelling, *Ensemble Modeling for Analysis of Cell Signaling Dynamics*, Nature Biotechnology 25(9), 2007.

[15] T.G. Robertazzi and S.C. Schwartz, An Accelerated Sequential Algorithm for Producing D-Optimal Designs, SIAM Journal on Scientific and Statistical Computing, SIAM, 10:341-358, 1989.
