# Supplementary figures and images for: Near-optimal experimental design for model selection in systems biology
Source: Bioinformatics. 2013 Jul 29;29(20):2625–32. doi: 10.1093/bioinformatics/btt436 (PMC3789540; doi:10.1093/bioinformatics/btt436)

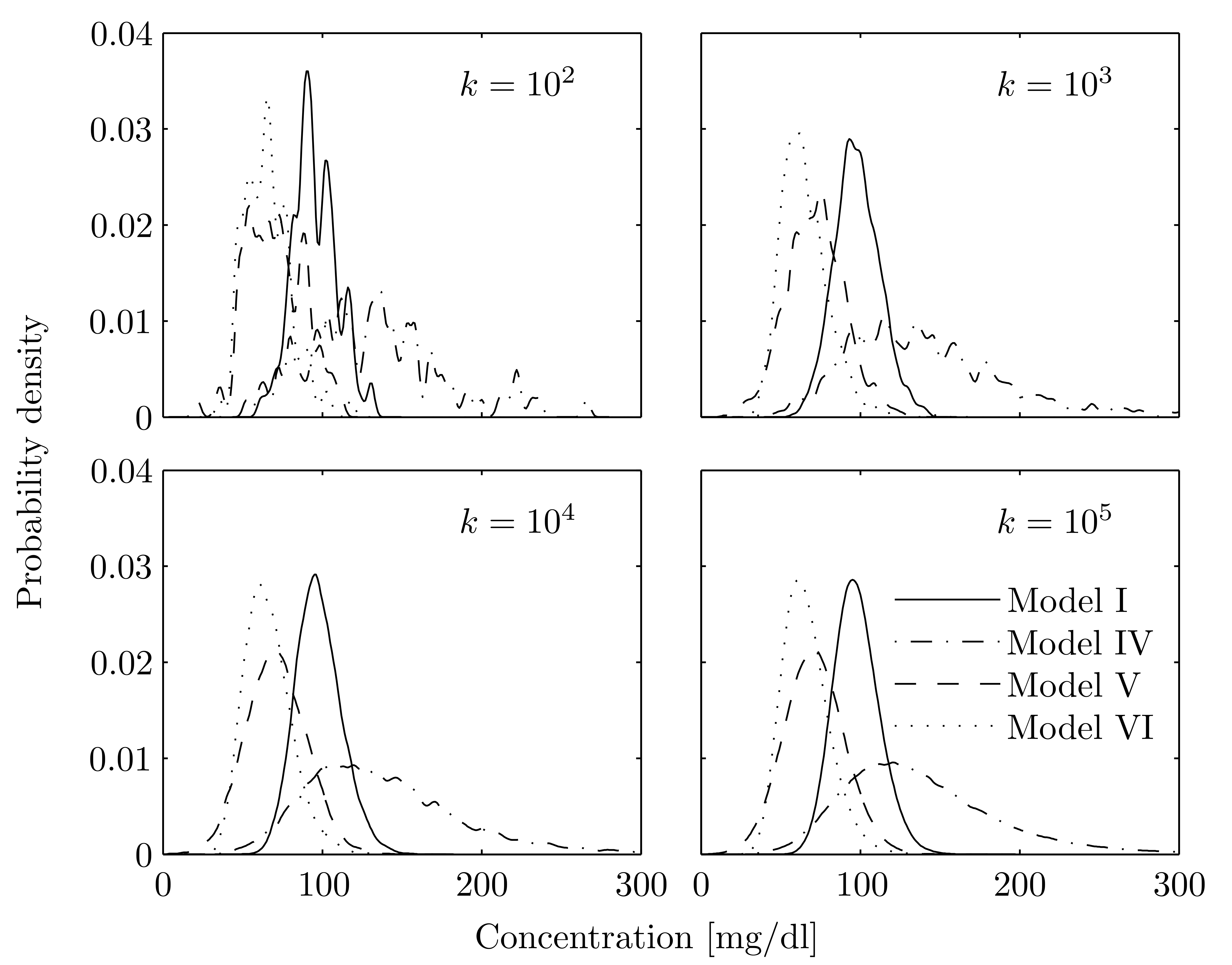

Supplement: Supplementary Data [file supp_btt436_FIG_S1.png]
